# Statistical characteristics of reset switching in Cu/HfO_2_/Pt resistive switching memory

**DOI:** 10.1186/1556-276X-9-694

**Published:** 2014-12-23

**Authors:** Meiyun Zhang, Shibing Long, Guoming Wang, Ruoyu Liu, Xiaoxin Xu, Yang Li, Dinlin Xu, Qi Liu, Hangbing Lv, Enrique Miranda, Jordi Suñé, Ming Liu

**Affiliations:** Lab of Nanofabrication and Novel Device Integration, Institute of Microelectronics, Chinese Academy of Sciences, Beijing, 100029 China; Departament d’Enginyeria Electrònica, Universitat Autònoma de Barcelona, Bellaterra, 08193 Spain

**Keywords:** RRAM, Statistics, Conductive filament, Weibull model, Thermal dissolution

## Abstract

A major challenge of resistive switching memory (resistive random access memory (RRAM)) for future application is how to reduce the fluctuation of the resistive switching parameters. In this letter, with a statistical methodology, we have systematically analyzed the reset statistics of the conductive bridge random access memory (CBRAM) with a Cu/HfO_2_/Pt structure which displays bipolar switching property. The experimental observations show that the distributions of the reset voltage (*V*_reset_) and reset current (*I*_reset_) are greatly influenced by the initial on-state resistance (*R*_on_) which is closely related to the size of the conductive filament (CF) before the reset process. The reset voltage increases and the current decreases with the on-state resistance, respectively, according to the scatter plots of the experimental data. Using resistance screening method, the statistical data of the reset voltage and current are decomposed into several ranges and the distributions of them in each range are analyzed by the Weibull model. Both the Weibull slopes of the reset voltage and current are demonstrated to be independent of the on-state resistance which indicates that no CF dissolution occurs before the reset point. The scale factor of the reset voltage increases with on-state resistance while that of the reset current decreases with it. These behaviors are fully in consistency with the thermal dissolution model, which gives an insight on the physical mechanism of the reset switching. Our work has provided an inspiration on effectively reducing the variation of the switching parameters of RRAM devices.

## Background

Resistive random access memory (RRAM), making full use of the reversible resistive switching (RS) effect of transition metal oxide to realize information storage, has been considered as a promising technology for high-density nonvolatile memory [1–4]. Due to its easy fabrication, promising performances, and feasibility of 3-D arrays, RRAM device, with a simple metal-insulator-metal (MIM) structure, has attracted considerable attention recently [5, 6]. A majority of works have focused on exploring the underlying switching mechanism for most transition metal oxide materials in set and reset processes [7–11]. Generally, the formation and rupture of a tiny conductive filament (CF) in the metal oxides is proposed to explain the resistive switching phenomena between a high-resistance state (HRS) or off-state and a low-resistance state (LRS) or on-state. Oxygen vacancies as well as metal ions are widely accepted as playing a dominant role in the formation and disruption of filament under the influence of external stress [12]. However, the size and location of CF in the set process and the extent of the CF dissolution during the reset process display random behaviors in RRAM devices, which causes the formation and rupture of the CF intrinsically stochastic [13] and results in the variation of the switching parameters and negatively affects the commercial application of RRAM [14–17]. Thus, studying the statistical characteristics of the switching parameters and deepening the understanding of the underlying physical mechanism behind the RS statistics are beneficial to the effective control and trustful forecast of the memory performance and reliability [18–23].

In this letter, we have investigated the reset statistical characteristics of the conductive bridge random access memory (CBRAM) device based on a Cu/HfO_2_/Pt structure connected to a transistor. The experimental results show that the reset voltage increases with on-state resistance and the reset current decreases with it, which can be well explained by the thermal dissolution model. Since the on-state resistance has strong influence on the reset switching parameters, the resistance screening method is employed to decompose the resistance into several ranges. The distributions of the reset voltage and current studied in different resistance ranges are compatible with the Weibull model. The Weibull slopes of reset voltage and current have nothing to do with the on-state resistance. The scale factor of the reset voltage linearly increases with the on-state resistance while that of the reset current decreases with it in linearity, respectively. These results are all consistent with the thermal dissolution model. Our work is of great significance on the deep understanding of the switching mechanism and the improvement of the uniformity of RRAM devices.

## Methods

**Figure 1:**
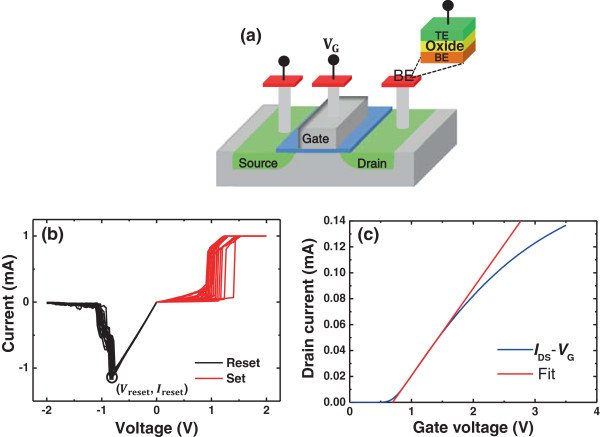
**Characteristics of the RRAM device and the transistor. (a)** The schematic of 1T1R structure. **(b)** Typical *I*-*V* curves of the Cu/HfO_2_/Pt device in set and reset cycles. The reset points have been notated by *V*_reset_ and *I*_reset_. **(c)** The transfer curve of the N^+^ transistor. The intrinsic parameters of the transistor (including *Wu*_n_ *C*_ox_/2 *L* and *V*_T_) are abstracted from the slope and the intercept of the fitting line, so the soure-drain resistance is obtained from calculation.

Figure 1a shows the fabricated 1T1R (one transistor and one RRAM cell) structure with which the RS statistics of the Cu/HfO_2_/Pt RRAM device are investigated. N^+^ type transistor was made up by standard 0.13 μm CMOS process of SMIC. Cu plug connected to the drain of the transistor was flattened by the chemical mechanical polished (CMP) and is used as the bottom electrode (BE) of our RRAM cell. A HfO_2_ RS layer was deposited by ion beam sputtering with a thickness of 6 nm on the Cu plug. Pt top electrode (TE) was then prepared by e-beam evaporation and patterned by lift-off process. The transistor in the 1T1R structure is used as the current compliance in the forming and set operation for RRAM cell to prevent the hard dielectric breakdown of the HfO_2_ layer and the overshoot of the current [24]. The electrical characteristics of the device were measured by Agilent B1500A Semiconductor Device Parameter Analyzer (Agilent Technologies, Inc., Santa Clara, CA, USA). The *I*-*V* curves in the 4,000 continuous set/reset cycles for the RRAM cell are tested under the DC voltage sweep mode. During the measurement, the voltage sweeping was applied on the source terminal in the set operation and on the drain terminal during the reset process, with the value of the voltage ramped from 0 to 2 V. The gate bias voltage of the transistor was set up as 2.5 V for the set operation to maintain a source-drain current of 1 mA to acquire an excellent current compliance and to avoid the RRAM cell being damaged by the current overshoot. In the reset operation, the gate bias was 3.3 V to guarantee that the CF is completely ruptured.

## Results and discussion

Figure 1b presents several *I*-*V* curves of the Cu/HfO_2_/Pt RRAM device. The metal-CF-type Cu/HfO_2_/Pt devices are operated in a bipolar mode. The point with the maximum value of current is defined as the reset point at which the voltage and current are recorded as the *V*_reset_ and *I*_reset_. We find that after the reset point, a series of current jumps occur during the reset process and the device finally switches to HRS. Through linear fitting to the reset *I*-*V* curve at the low-voltage region before the reset point, the on-state resistance of the 1T1R structure (*R*_on___total_) is obtained, which is a sum of the LRS resistance of RRAM cell (*R*_on_) and the source-drain resistance of the transistor (*R*_DS_). *R*_on_ is then got through correcting *R*_on___total_ by *R*_DS_. *R*_DS_ is usually in the order of several hundreds of ohms, which is comparable to *R*_on_, so it should not be neglected during the reset process of RRAM device in 1T1R structure. Figure 1c shows a tested transfer characteristic curve (*I*_DS_ - *V*_G_ curve) of the transistor with the source-drain voltage fixed to be 0.05 V. Through fitting the curve according to the output characteristic of the transistor with the equation , the intrinsic values of *Wu*_n_*C*_ox_/2 *L* and *V*_T_ are obtained, where *u*_n_ is the electronic mobility, *W* is the gate width, *L* is the gate length, and *C*_ox_ is the capacitance of the gate oxide. Based on the output characteristic curve (*I*_DS_ - *V*_DS_ curve) of the transistor in the reset operation under *V*_G_ = 3.3 V, the source-drain resistance is available to be 300 Ω in average according to *R*_DS_ = *V*_DS_/*I*_DS_. Here, *I*_DS_ is the measured current flowing through the transistor and the RRAM device, and *V*_DS_ is calculated from the above equation using the abstracted *Wu*_n_*C*_ox_/2 *L* and *V*_T_ value and the measured *I*_DS_ values.

**Figure 2:**
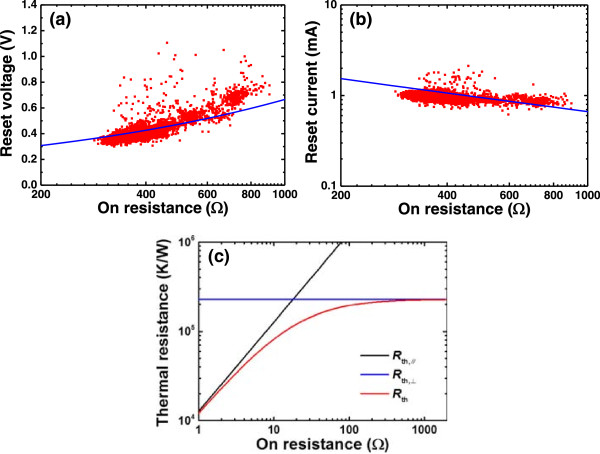
**Scatter plots of** ***V***_**reset**_ **and** ***I***_**reset**_ **and dependence of the calculated** ***R***_**TH**_ **on** ***R***_**on**_ **.** The dependence of the *V*_reset_ **(a)** and *I*_reset_ **(b)** as a function of *R*_on_. *V*_reset_ increases and *I*_reset_ decreases with *R*_on_, respectively, which are well fitted by the thermal dissolution model (blue lines) with *T*_reset_ = 400 K, *R*_TH,⟂_ = 2.3 × 10^5^ K/W. **(c)** The dependence of calculated thermal resistance on the CF resistance. *R*_TH,⟂_ is considered as being constant with a value of 2.3 × 10^5^ K/W.

The relationships of *V*_reset_ and *I*_reset_ with *R*_on_ are studied with a statistical method. Figure 2a,b shows the scatter plots of *V*_reset_ and *I*_reset_ as a function of *R*_on_ in the 4,000 continuous set and reset cycles. As can be seen, the spread of *V*_reset_ and *I*_reset_ is slightly wide and *V*_reset_ increases while *I*_reset_ decreases with *R*_on_. These characteristics can be accounted for by the thermal dissolution model [21–23, 25–28] which assumes that reset is determined by the diffusion of the conductive defects. In Cu/HfO_2_/Pt RRAM device, the conductive defects are mainly the Cu metal atoms or ions. When the local CF temperature reaches a critical value *T*_reset_, the conductive defects begin to diffuse out of the CF and then reset occurs. Considering the balance between Joule dissipation and heat evacuation, the local temperature of the CF can be calculated by the basic equation  where *T*_0_ is the operation temperature, *V*_reset_ is the voltage dropped on the CF at the reset point, and *R*_TH_ is the thermal resistance describing heat dissipation from the CF to the environment. The thermal resistance *R*_TH_ can be divided into two components in parallel, the parallel resistance (*R*_TH,∥_) and the perpendicular resistance (*R*_TH,⟂_). Their relation is described by *R*_TH_ = *R*_TH,∥_*R*_TH,⟂_/(*R*_TH,∥_ + *R*_TH,⟂_), where *R*_TH,∥_ = *R*_on_/(8*LT*_reset_) according to the Wiedemann-Franz (WF) law and *L* = 2.45 × 10^- 8^ WΩK^- 2^ is the Lorentz number [23]. The two components respectively describe the heat diffusion along the CF and from the CF surface to the surrounding oxide. Figure 2c shows the dependence of the calculated *R*_TH_ on *R*_on_. At the low-resistance region, the value of *R*_TH_/*R*_on_ is roughly constant and then when *R*_on_ increases, the ratio will not be constant anymore. Using the above relations, we can get the theoretical fittings for the experimental relationships between *V*_reset_, *I*_reset_, and *R*_on_ through choosing appropriate parameters of *T*_reset_ and *R*_TH,⟂_. By the theoretical fitting, *V*_reset_ is proved to increase with *R*_on_, which is consistent with our experimental results shown in Figure 2a. In this work, the on-state resistance is comparatively high, as compared with the reports in [21, 22], so *V*_reset_ increases with *R*_on_. This increase trend is similar to the *V*_reset2_ - *R*_reset2_ relation reported in [23]. Since the CF behaves like a metallic conductor before the reset point [29], *I*_reset_ is inversely proportional to *R*_on_ as presented in Figure 2b.

**Figure 3:**
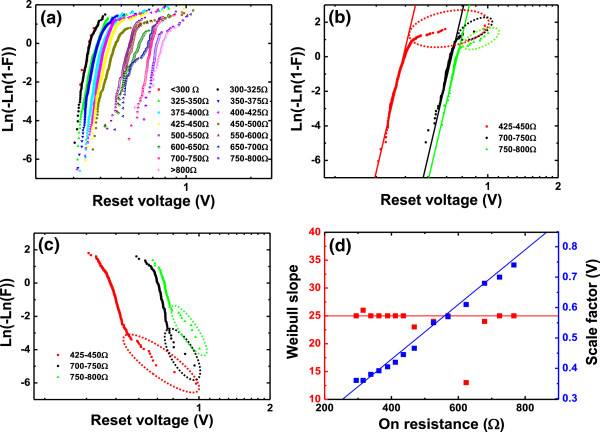
**Experimental distributions of** ***V***_**reset**_ **as a function of** ***R***_**on**_ **. (a)** Experimental distributions of *V*_reset_ in different *R*_on_ ranges in Weibull plot. **(b)** Distributions of *V*_reset_ with fitting lines in three resistance ranges. The fitting lines show that the experimental distributions are roughly compatible with Weibull distributions. **(c)** The Gumbel distribution of *V*_reset_ in the same three resistance ranges as in **(b)**. A small part of data fall into the circles, indicating that a small proportion of data belong to the tailing region of the distributions in **(b)**. **(d)** The dependence of the Weibull slope and scale factor of *V*_reset_ distribution on *R*_on_. The Weibull slope remains constant and the scale factor increases linearly with *R*_on_.

To further study the details of the relationship of *V*_reset_ and *I*_reset_ as a function of *R*_on_, the resistance screening method is utilized through which *R*_on_ is reasonably divided into several ranges. The statistical distributions of *V*_reset_ and *I*_reset_ in different ranges decomposed by the resistance method are studied in detail. Figure 3a shows the experimental distributions of *V*_reset_ in different *R*_on_ ranges in the Weibull plot. Figure 3b shows the typical distributions of *V*_reset_ in three different resistance ranges with linear fitting lines. We can conclude that the Weibull distribution can be used to well describe the experimental distributions of *V*_reset_ and *I*_reset_ of the Cu/HfO_2_/Pt device. In Figure 3a,b, we find a high-percentile tail exists in the distribution in each *R*_on_ range, which is deviated from the standard Weibull distribution which is a straight line in the Weibull plot. Figure 3c shows the same three distributions in Figure 3b in the Gumbel plot, which can show more clearly the high-percentile tail region in the Weibull plot. As shown in Figure 3c, it can be seen that the experimental data in the high-percentile tail region in Figure 3b are just a very small part in the whole distribution in each range. Fitting all the experimental results in Figure 3a by linear Weibull distribution, the shape factors (i.e., the Weibull slopes) and the scale factors of *V*_reset_ distributions can be obtained. Figure 3d shows the dependence of the shape factor (*β*_V_) and scale factor (*V*_63 %_) of *V*_reset_ distributions on *R*_on_. We can find that the Weibull slope of *V*_reset_ distribution remains constant and the scale factor of the *V*_reset_ distributions increases with *R*_on_.

**Figure 4:**
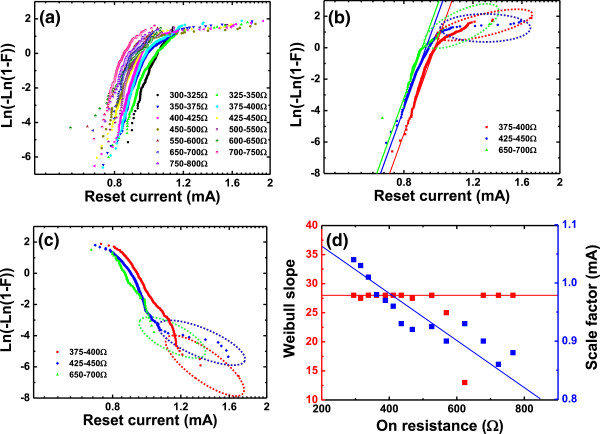
**Experimental distributions of** ***I***_**reset**_ **as a function of** ***R***_**on**_ **. (a)** Experimental distributions of *I*_reset_ in different *R*_on_ ranges in Weibull plot. **(b)** Distributions of *I*_reset_ with fitting lines in three *R*_on_ ranges. The fitting lines show that the experimental distributions are roughly compatible with Weibull distributions. **(c)** The Gumbel distribution of *I*_reset_ in the three *R*_on_ ranges same as those in **(b)**. A small part of data fall into the circles, indicating that a small proportion of data belong to the tailing region of the distributions in **(b)**. **(d)** The dependence of the Weibull slope and scale factor of *I*_reset_ distributions on *R*_on_. The Weibull slope stays constant, and the scale factor decreases linearly with *R*_on_.

Analogous to the study of the reset voltage, the reset current are analyzed in the same way. Figure 4a,b shows the Weibull distributions of *I*_reset_ in different *R*_on_ ranges with a certain high-percentile tails. These tails are also demonstrated to occupy only a small proportion by Gumbel distributions, as shown in Figure 4c. Through the linear fitting to the standard Weibull distributions, the abstracted Weibull slope and scale factor of the reset current distributions as a function of *R*_on_ are illustrated in Figure 4d. The Weibull slope of the reset current stays constant, and the scale factor decreases with *R*_on_.

**Figure 5:**
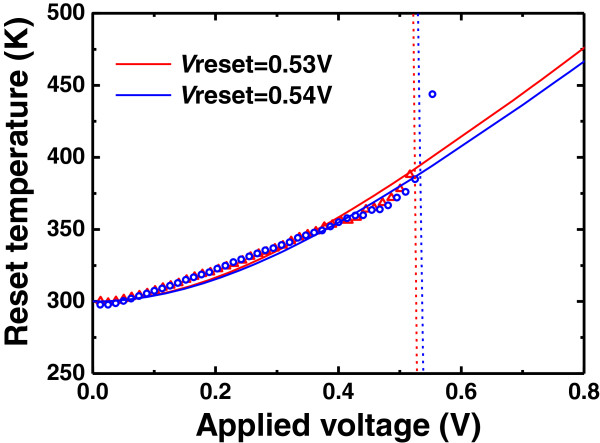
**Experimental (symbols) and theoretical reset temperature (lines) of two reset cycles in the Cu/HfO**_**2**_ **/Pt device.** The dashed lines indicate the reset voltage dropped on the RRAM cell, corresponding to the maximum current, i.e., the reset point. The experimental and theoretical curves nearly coincide before the reset point, so the reset point represents the starting point of the CF dissolution.

In our previous work [21], an analytical model based on the thermal dissolution model has been proposed for the unipolar reset statistics. Implementing this model into the experimental statistics of our Cu/HfO_2_*/*Pt RRAM device, the abstracted Weibull slopes of *V*_reset_ and *I*_reset_ distributions, which are both independent on *R*_on_, indicate that the reset point corresponds to the initial step of the CF dissolution in this device and there is no structural degradation before the reset point. This result can be evidenced by comparing the experimental and theoretical maximum CF temperature curves detailedly described in [22]. Figure 5 shows the experimental and theoretical reset temperature curves for two cycles of our Cu/HfO_2_*/*Pt device. In Figure 5, before the reset point, the two types of curves are exactly in coincidence, which indicates that the reset point just corresponds to the starting point of the CF dissolution. In consequence, the statistical results of Cu/HfO_2_*/*Pt RRAM device are compatible with the thermal dissolution model.

The above experiment and thermal dissolution model results both demonstrated that the statistical spread of reset parameters *V*_reset_ and *I*_reset_ are intrinsically limited by the on-state resistance *R*_on_, so controlling the value and distribution of *R*_on_ is very critical to acquire high uniformity of reset switching. In the previous works, some methods have been proposed to reduce the variation of the off-state resistance (*R*_off_) and on-state resistance (*R*_on_), through which the uniformity of the set and reset switching parameters also have been improved and the set and reset events are controlled in certain ranges. These methods include: 1) doping impurities in the switching layer [30], selecting the electrode materials [31], and inserting interface layers [32] so as to effectively control the concentration and distribution of defects such as charge traps, metal ions, and oxygen vacancies etc.; 2) introducing the electric field-concentrating initiators (e.g., nanocrystals) on the bottom electrode to enhance the local electric field and reduce the random growth of filaments [16]; and 3) utilizing optimized operation methods [33]. In our recent work, we have also paid attention to the new pulse operation with regard to its role in improving the switching uniformity. Different from the traditional single pulse operation method in which only one wide pulse is applied in each switching cycle, a novel width/height-adjusting pulse operation method is proposed for RRAM. This method utilizes a series of pulses with the width or height increased gradually until a set or reset switching process is completely finished and no excessive stress is produced. The new operation method can exactly control the final resistance and significantly improve the uniformity, stability, and endurance of RRAM device. Additionally, we are also focusing on optimizing the device structure by etching the substrate into a cone shape on which the RRAM device is fabricated. The optimized structure can control the formation and rupture of the conductive filament in the oxide layer at the tip of the cone shape due to the high-generated electric field in this position. Thus, the variation of the switching parameters can be significantly reduced and the uniformity of these parameters will be improved.

## Conclusions

In summary, we have studied the reset statistics of the CBRAM device with a Cu/HfO_2_*/*Pt structure. The experimental results show that the reset voltage increases with on-state resistance and reset current decreases with it. The distributions of the reset voltage and current observed in different resistance ranges divided by ‘resistance screening method’ are compatible with the Weibull model. The Weibull slopes of reset voltage and current are constant, independent of the on-state resistance. The scale factors of the reset voltage increases and that of the reset current decreases with the on-state resistance in linearity, respectively. These results are in agreement with the thermal dissolution model. Our work is helpful in revealing the physics of the switching mechanism and giving guidelines to improve the uniformity of RRAM devices.
